# Pathophysiology of subchondral bone erosions in rheumatoid arthritis

**DOI:** 10.1186/ar3721

**Published:** 2012-03-08

**Authors:** Georg Schett

**Affiliations:** 1 Universitätsklinikum Erlangen, Medizinische Klinik 3, Rheumatologie & Immunologie, Erlangen, Germany

## 

The presentation describes the novel insights on the interaction between the immune system and bone over the last 10 year, and explains molecular and cellular interactions as well as their clinical implications.

Bone is subject to a continuous remodeling process which allows an ideal adaptation to the individual demands throughout life. This remodeling process is based an interplay between bone forming osteoblasts and the bone resorbing osteoclasts. Several conditions alter this balance, among them the drop of estrogens levels after menopause is the most well known factor, which leads to enhanced bone resorption and bone loss. Interestingly, all different forms of inflammatory diseases such as rheumatoid arthritis, systemic lupus erythematosus and inflammatory bowel disease also interfere with the bone remodeling process and precipitate bone loss and increased fracture risk.

To study this interaction between immune activation/inflammation and the skeletal system has become a novel research discipline called osteoimmunology. This field has gained insights into the mechanism of inflammatory bone loss, in particular of how inflammatory cytokines like TNF-alpha, IL1 or IL6 foster bone resorption and inhibit bone formation, resulting in an imbalance of bone homeostasis and thereby precipitating bone loss. Therapeutic inhibition of cytokines has yields profound changes architecture in arthritis patients and protects the bone and in part also the articular cartilage. Inflammatory cytokines particularly enhance bone loss by activation of osteoclast differentiation factors such as MCSF and RANK ligand and by molecules which interfere with bone formation such as DKK1 and sclerostin.

The insights into molecular regulation of bone remodelling by the immune system are thus of key interest in better understanding inflammatory diseases such as arthritis and to shape novel therapeutic concepts. Research in the field of osteoimmunology has a strong translational approach and directly affects patients suffering from inflammatory diseases, in particular arthritis, in aiming to prevent skeletal damage and loss of physical function.

